# Oral Drug Delivery via Intestinal Lymphatic Transport Utilizing Lipid-Based Lyotropic Liquid Crystals

**DOI:** 10.3390/liquids3040029

**Published:** 2023-11-20

**Authors:** Linh Dinh, Bingfang Yan

**Affiliations:** Division of Pharmaceutical Sciences, James L. Winkle College of Pharmacy, University of Cincinnati, Cincinnati, OH 45229, USA

**Keywords:** lyotropic liquid crystals, cubosomes, hexosomes, oral delivery systems, sustained release, intestinal lymphatic transport, chylomirons, M cells

## Abstract

Lyotropic liquid crystals (LLCs) are liquids that have crystalline structures. LLCs as drug delivery systems that can deliver hydrophobic, hydrophilic, and amphiphilic agents. Due to their unique phases and structures, LLCs can protect both small molecules and biologics from the gastrointestinal tract’s harsh environment, thus making LLCs attractive as carriers for oral drug delivery. In this review, we discuss the advantages of LLCs and LLCs as oral formulations targeting intestinal lymphatic transport. In oral LLC formulations, the relationship between the micelle compositions and the resulting LLC structures as well as intestinal transport and absorption were determined. In addition, we further demonstrated approaches for the enhancement of intestinal lymphatic transport: (1) lipid-based LLCs promoting chylomicron secretion and (2) the design of LLC nanoparticles with M cell-triggered ligands for targeting the M cell pathway. In this review, we introduce LLC drug delivery systems and their characteristics. Our review focuses on recent approaches using oral LLC drug delivery strategies targeting the intestinal lymphatic system to enhance drug bioavailability.

## 1. Introduction

**Figure 1.**
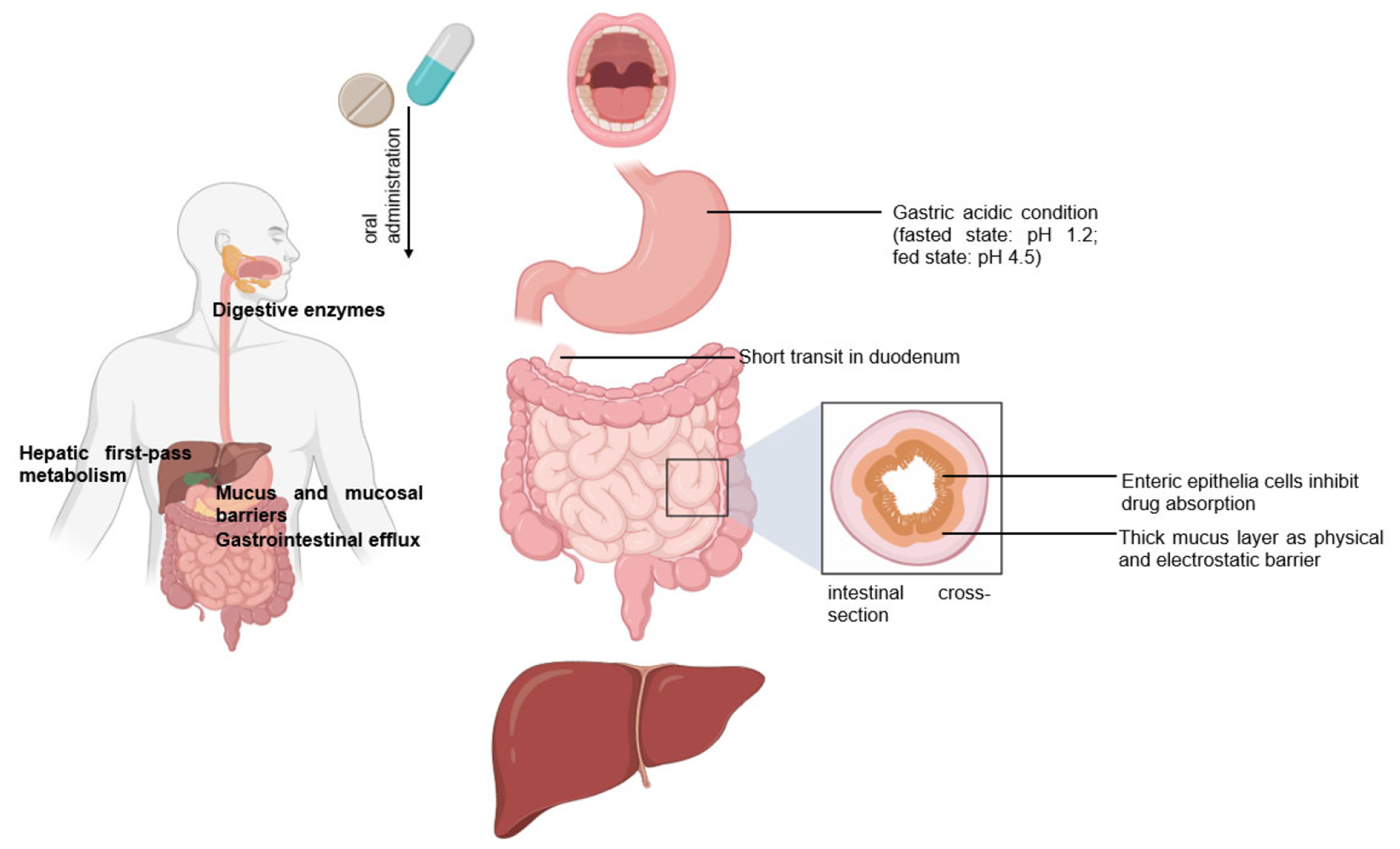
Following the oral administration of a therapeutic, the drug travels from the mouth to its site of action. The drug enters the digestive tract where it faces challenges.

Peroral is the most common delivery route for drug administration [1–3]. Oral dosing is the most preferred by patients and convenient to handle for healthcare providers because taking medicines by mouth is non-invasive and requires no sterility constraints compared to injectables [2–4]. However, an orally administered drug formulation must survive the gastrointestinal tract’s harsh environment, gastric acidic conditions (pH 1.2), the presence of digestive enzymes, the mucus and mucosal barriers, the enteric epithelia whose tissue morphology inhibits drug absorption, and the hepatic metabolism for local and/or systemic therapy (Figure 1) [1–5]. Notably, hepatic first-pass metabolism, also known as drug pre-systemic exposure, heavily reduces the pharmacokinetic effects of drugs [2,4,5]. Thus, the oral bioavailability of many small molecule active pharmaceutical ingredients (API) (of biopharmaceutical classification system (BCS) III (high solubilitypoor permeability) and IV (poor solubilitypoor permeability) drug classes) is often very poor, and the oral delivery of biologics remains a great challenge [2,5].

**Figure 2.**
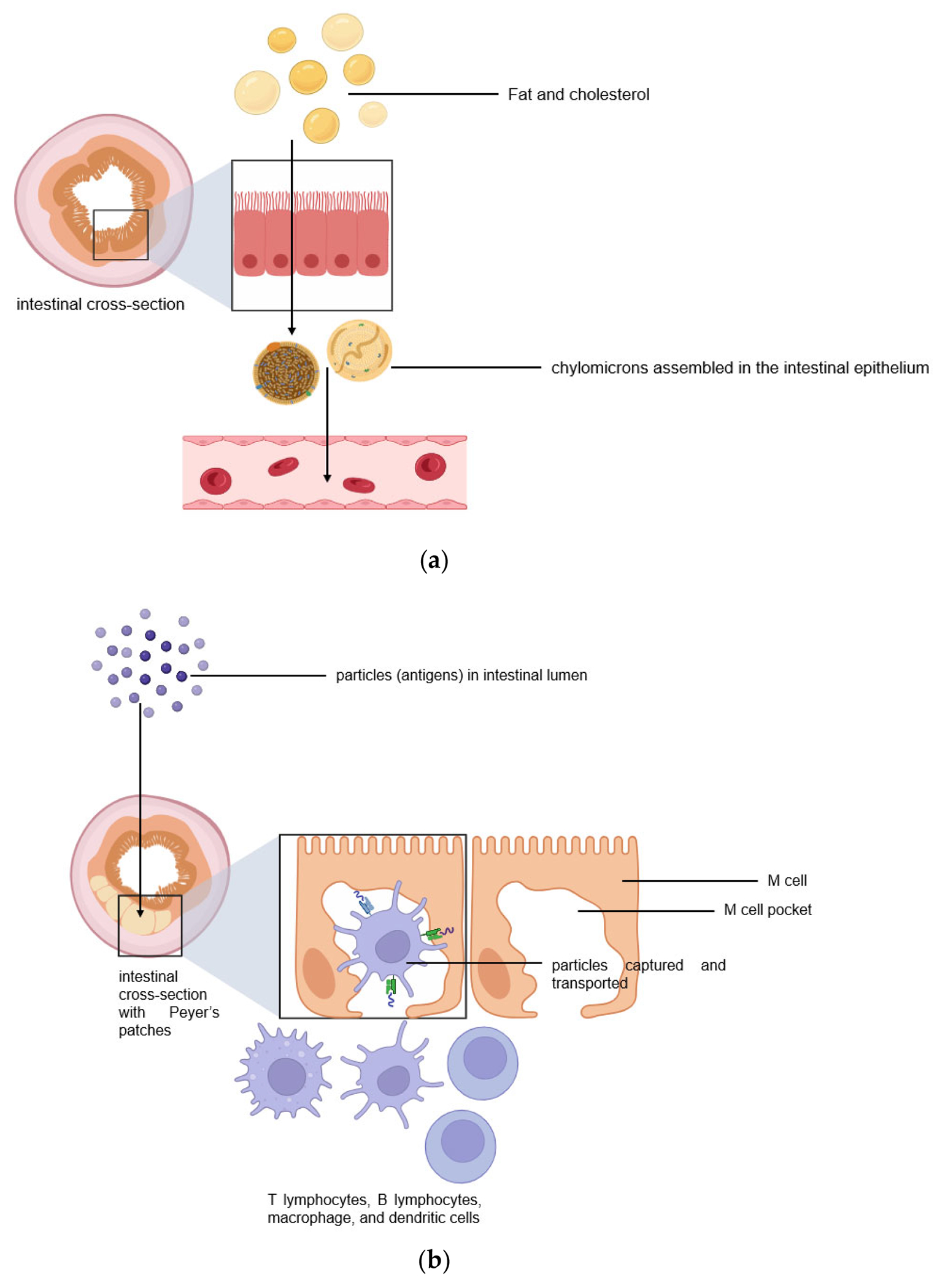
Two pathways for efficient intestinal lymphatic transport: (**a**) the chylomicron pathway and (**b**) the M cell pathway.

The intestinal lymphatic pathway can be considered as the ultimate solution for drug transport to avoid first-pass metabolism [1,2,5–7]. The intestine absorbs fat both through passive diffusion and protein-facilitated transfer [2,5–8]. The exogenous lipoprotein pathway utilizing chylomicronsthe fat carriers assembled in the intestinal epithelium, is responsible for transferring the food-derived lipids and fat-soluble vitamins to the cells [5–8]. Therefore, by targeting the chylomicrons in enterocytes, lipid-based drug formulations can be absorbed from the intestine via lymphatic transport through the chylomicron pathway, and they enter the bloodstream through the thoracic lymph duct (Figure 2a). Another promising lymphatic pathway for oral drug delivery is the microfold cells (M cells) channel [5,7,9]. The epithelium covering mucosa-associated lymphoid tissues is where M cells are typically found. In the ileum, M cells mediate the interface between the lumen and the lymphatic system by capturing and transporting pathogen-like particles. To initiate an immune response, M cells actively deliver luminal particles and antigens to intestinal dendritic cell subsets lying under lymphoid follicles (Figure 2b). The lymphatic vessels are closely associated with blood vessels, and they are well-distributed throughout the intestinal wall and lined with endothelial cells that are highly permeable to lipophilic molecules. Herein, we discuss several studies that have engineered drug delivery vehicles so that lymphatic vessels and immune cells in the intestines can absorb them more efficiently.

**Figure 3.**
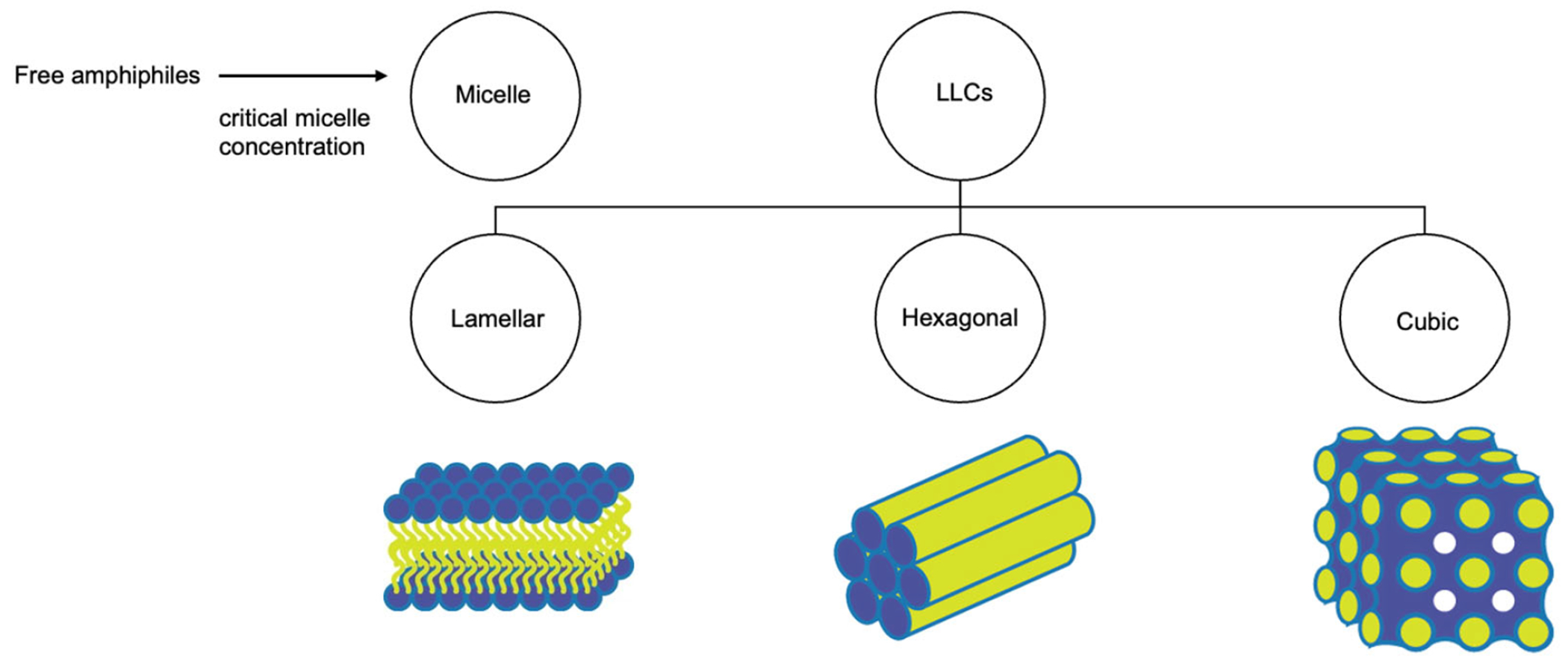
Structural classification of LLCs.

**Figure 4.**
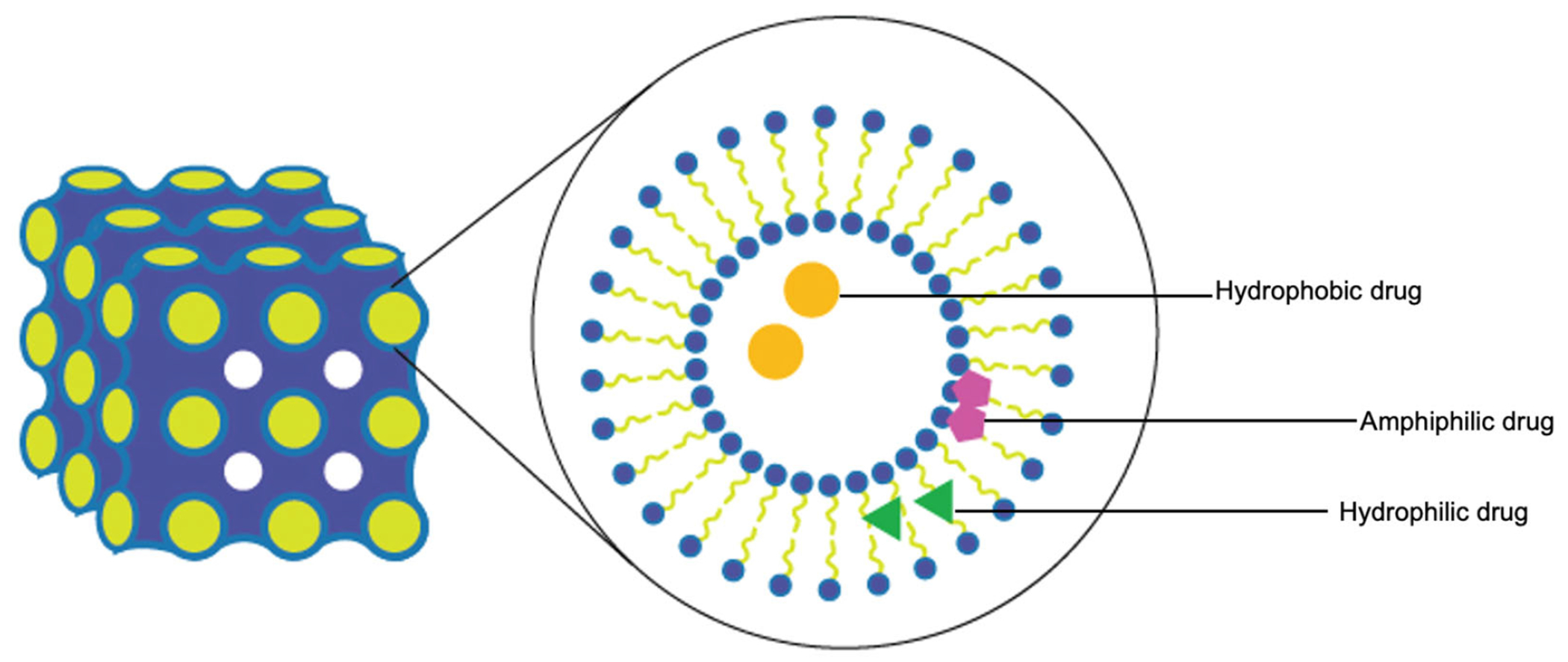
Cubosomes encapsulate hydrophobic, hydrophilic, and amphiphilic model drugs.

Lyotropic liquid crystals (LLCs) result when amphiphiles (amphiphilic mesogens) dissolve into suitable solvent-forming solutions that flow like liquids but still have ordered crystalline structures of solid crystals [10,11]. The well-defined, thermodynamically stable structures of LLCs such as lamellar, hexagonal, and cubic (Figures 3 and 4) are formed by the self-assembly of amphiphilic lipids due to hydrophobic forces. By dispersing these mesophase structures into water in the presence of stabilizers, they can be transformed into liposomes, hexosomes, and cubosomes, collectively known as LLC drug delivery systems [11]. In recent years, due to their unique structures offering matrices for prolonged drug release as well as their amphipathic nature for the encapsulation of hydrophobic, amphiphilic, and hydrophilic molecules of various molecular weights, LLC drug delivery systems have drawn significant attention as one of the advanced systems in the field of colloidal dispersions [11–13]. Figure 4 shows how a hydrophobic drug, a hydrophilic drug, and an amphiphilic drug are encapsulated in cubosomes. Many lipids that form LLCs are biocompatible and biodegradable and are applicable to the oral route of administration [12,14]. Intestinal lymphatic absorption is a pathway that an LLC drug delivery system may follow to gain access to the systemic circulation after its oral administration. A lipid-based LLC drug delivery system targeting the intestine can enhance intestinal lipid fluidity.

In this review, we introduce LLC drug delivery systems, their characteristics, and their effects as oral formulations. Our review focuses on recent approaches using oral LLC drug delivery strategies targeting the intestinal lymphatic system to enhance drug bioavailability and reduce first-pass metabolism.

## 2. LLC Drug Delivery Systems for Efficient Intestinal Lymphatic Targeting

### Preparation and Characterization of Oral LLC Drug Delivery Systems

Table 1 presents the compositions and phases of LLC systems. An LLC system includes water (aqueous phase), amphiphilic mesogen (lipid phase), and surfactant. The mesophase is often determined by the hydrophobicity and hydrophilicity of the system. When the concentration of micelles dispersed in water is above the critical aggregation concentration (CMC), micelles are forced to pack into a structure (micellar cubic phase—discontinuous cubic phase) [12,15]. The lamellar phase consists of lipid bilayers with hydrophilic heads (outer layer) and hydrophobic tails (inner layer). In water, these lipid bilayers are arranged linearly with water channels. Small changes in temperature and concentration can cause transitions between mesophases (Figure 4). Bicontinuous cubic and hexagonal phases are made of a 3D network of bicontinuous lipid bilayers arranged in a pattern of infinite periodic minimal surfaces (bicontinuous cubic phase) and in cylinders (hexagonal phase). These 3D networks have two distinct, continuous, non-intersecting, hydrophilic sections, and hydrophobic components are placed within. The cubic and hexagonal phase dispersions are especially advantageous for oral delivery because their structure could protect the drug in the gastrointestinal tract’s harsh environment [16]. Moreover, LLCs with a hydrophilic surface can easily traverse the water layer and contact the endothelial cell layer. Cubosomes were reported to have the ability to penetrate through the endothelial cell membrane [17,18].

Cubosomes and hexosomes are typically prepared for drug delivery by forming dispersions using energy input methods. Glyceryl monooleate and phytantriol are the two most used lipids to form cubic and hexagonal phases (Table 1). Surfactants and stabilizers are often incorporated into the preparation to maintain its colloidal stability. The “gold standard” steric stabilizer for the preparation of LLC is poloxamer 407 (Pluronic^®^ F127 (BASF, Ludwigshafen, Germany), polyethylene-polypropylene glycol, hydrophilic–lipophilic balance (HLB) = 18) (Table 1) [19,20]. Poloxamer 407 kinetically stabilizes the formation of LLC by working on the interfacial tension, because it is composed of polyethylene oxide (PEO)-polypropylene oxide-PEO block copolymer that contains both hydrophilic and hydrophobic parts. The hydrophobic part interacts with the lipid bilayer, while the hydrophilic part faces the aqueous portion [21]. Bile salts, amphiphilic proteins, block polymers, other non-ionic surfactants, and other types of stabilizers (PEGylated lipids and customized lipid–copolymer (lipidic polymers)) including polysorbate 80, Cremophor^®^, and Labrasol^®^ (BASF, Ludwigshafen, Germany), were investigated for their ability to stabilize LLCs [19,22]. The choice of surfactants for oral LLC systems was based on their required ability to protect APIs from premature metabolism by liver enzymes. Their high HLB value indicates their tendency to solubilize lipid (low HLB) in water, thereby forming the desired structure required to encapsulate a drug. For surfactants with HLB values > 6, cholesterol addition is recommended to form bilayered vesicles in the case of lamellar LLCs [23]. A chosen stabilizer is also required to have the bioactivity to inhibit the function of P-glycoprotein (P-gp) in the intestine, thereby increasing intestinal absorption [24]. Moreover, it was suggested that the PEO blocks of Poloxamer 407, covering the outer surfaces of formed cubosomes and/or hexosomes drain relatively faster into the lymphatic system, avoiding detection by lymph node macrophages, thus reaching the blood circulation and remaining there for a long period of time [25].

LLC drug delivery systems are characterized by their mesophase identification, drug release patterns, stability, and drug entrapment efficiency (EE) that is calculated as follows:

$$\text{EE}(\%)=\frac{\text{weight of drug entrapped into a LLC system}}{\text{total weight of drug added}},$$

For LLC formulations, EE and in vitro release are the most important characteristics. Both cubosomes and hexosomes can encapsulate the drug within the LLC system, and depending on the drug solubility, a water-insoluble drug can be found in the hydrophobic sections (surfactant hydrophobic parts and hydrophobic tails) and a water-soluble drug can be found in the hydrophilic sections (water channels) of the network. A general procedure to produce an LLC dispersion is to dissolve hydrophobic drugs in the oil phase with surfactants and then homogenize the oil phase mixture and water. Barauskas et al. compared different nanoparticle dispersions of self-assembled lipid mesophases and suggested that the release profile of hydrophobic drug-loaded cubosomes was significantly improved [26]. This can be explained by the fact that it is more difficult for hydrophobic drugs to escape from the LLC system compared to hydrophilic ones. The affinity of hydrophobic drugs with hydrophobic cores of reversed cubic phase is strong. Meanwhile, hydrophilic drugs trapped inside the LLC systems can flow out to the outer water through the water channels. Interestingly, amphiphilic drugs can be trapped in both hydrophilic and hydrophobic sections and along the interface of lipid and water (Figures 3 and 4). The drug release from an LLC drug delivery system is based on the principle of drug diffusion following the Higuchi diffusion kinetic model [11,12,32,33]. Studies have shown that the LLC structure and the nature of lipids could be utilized to control the drug release rate, but an initial burst release before a sustained drug release seems unavoidable [11,12,34]. Initial burst release phenomenon and drug release kinetics are independent and are not related to EE. An LLC product with a high drug loading may cause local or systemic toxicity due to an initial burst release [34].

An LLC’s mesophase behavior can be affected by the choice of surfactant and mesogen and by several other factors. On the other hand, LLC mesophase behavior affects the physiochemical characteristics, EE (cubosomes were reported to be better than hexosomes in most cases), and drug release patterns (cubic phase often released drug faster than hexagonal phase) of the LLC system [35–37]. It is important to identify the LLC phase, especially when the phase determines whether the drug has been incorporated into it. Mesophases are detected and characterized by methods mentioned in Table 2. Structural details of an LLC can be observed using transmission electron microscopy (TEM), but small-angle X-ray scattering (SAXS) and small-angle neutron scattering (SANS) can visualize the structure as well as the phase behavior of LLCs using lattice parameters of the phase. The morphology of an LLC can be determined using TEM, in which samples are drop-casted on a mesh grid. Traditional TEM, in which materials are dried and stained on carbon grids before being observed using a microscope, is not recommended because of dehydration. Cryogenic electron microscopy (Cryo-EM) has been used to obtain the high-resolution images of LLCs, enabling the direct visualization of the internal phase structure and size assessment without fixating or staining [38]. To analyze LLC mesophases, experiments that are used are complex, and more than one technique can be used. Experiments are often run at various concentrations of mesogens. As an LLC phase transition occurs, fluctuations in heat capacities and enthalpy can be measured using differential scanning calorimetry (DSC). Particle size and zeta potential values measured using dynamic light scattering are often determined for the stability tests and quality control of LLCs.

The physical properties, stability, and physiological behavior of LLCs are highly critical characteristics. The nanostructured LLCs were considered as the template for nanostructured biodegradable hydrogel networks [39]. The choice of materials and the concentration of mesogens dramatically affected the network diffusivity and permeability, thus leading to changes in the degradation rate of the lamellar phase. For nanostructured LLCs, being nanosized may facilitate the release of drug from the carriers, and no significant relation between the mean particle size value and the release rate as well as the degradation rate was observed.

## 3. The Intestinal Lymphatic Pathway and LLC Approaches for the Enhancement of Intestinal Lymphatic Transport

The main advantage of LLCs is related to their ability to encapsulate drugs as cubosomes and hexosomes. Cubic phased LLCs can easily and instantly transform into lamellar phase and vice versa. The in situ phase transition makes LLCs uniquely applicable to different routes of administration and organ-specific target applications. This study focuses on LLCs as a delivery system targeting the intestinal lymphatic system.

### 3.1. Enhancement of Chylomicron Transport

The intestinal lymphatic system is responsible for the absorption and transportation of lipids [2,5–8,46]. Lymphatic delivery of small molecules is achievable by nanoparticles, polymers, liposomes, or via in situ association with proteins, lipoproteins, and lymphotropic leukocytes [5–7,9,30,47,48]. Fat-soluble particles are absorbed by intestinal epithelial cells, released as chylomicrons, and transferred by lymphatic vessels [8,49]. Similarly, upon enterocytes, drug–lipoprotein complexes are transported from the intestinal lamina propria via the lymphatics when the drug is administered with lipids (from food or a formulation). Inspired by the conventional oral formulations of lipophilic prodrugs and co-administered oil–drug formulations promoting chylomicron secretion, lipid-based drug delivery systems have emerged to become the most common and efficient solution when it comes to the enhancement of chylomicron transport. As discussed above, to avoid the water and mucosal layers found within the small intestine limiting the transport of drugs into endothelial cells, bile salts, phospholipids, cholesterol, and other types of lipids were used to help promote the formation of colloidal vesicles. These lipid droplets (multilayered LLCs) help increase the solubilization of drugs, especially poor-water soluble ones, by solubilizing them into micelles with the lamellar vesicles formed by the lipid layers of LLCs, thus increasing the oral bioavailability of drugs [5]. Therefore, LLCs consist of an oil phase and a solubilized micellar phase during lipid digestion, enhancing both the solubility and bioavailability of drugs in the lumen. Tran et al. ‘s quercetin-loaded-in situ self-emulsifying drug delivery system enhanced the oral bioavailability of quercetin by improving its solubility and lymphatic absorption. In male Sprague Dawley rat models, the Caco-2 permeability of quercetin oil/in/water nano-emulsion improved significantly due to the chylomicron uptake in the lymphatic system [50]. Quercetin, a hydrophobic drug, was confirmed to be transported by chylomicrons. Quercetin-loaded lipid liquid crystalline systems were developed, and they may increase quercetin’s lymphatic transport in the small intestine [50–52]. It was concluded that glyceryl monooleate cubosomes possibly did not show any ability to alter the integrity of the Caco-2 cellular tight junctions [53]; thus, the lymphatic transport pathway of lipid-based LLC nanoparticles mainly occurs via chylomicrons.

While the formation of chylomicrons does not relate directly to the ordered lipid mesophases in the gastrointestinal tract, the link between the influence of lipid digestion on the fate of orally administered cubosomes and the enhancement of oral bioavailability has been deciphered [52–56]. Moreover, the nanosized LLC delivery systems were stable in the gastrointestinal tract and were permeable across the gastrointestinal epithelium [17,54]. According to Leesajakul et al., the long in vivo circulation time of drugs in cubosomes was because of the sustained behavior of multilayered lipid-based LLCs [57]. Glyceryl monooleate and oleyl glycerate-based cubosomes containing cinnarizine showed a high drug loading with an improved sustained release of a drug compared to that of a drug suspension [31]. Glyceryl monooleate-based LLCs showed both a sustained-released mucoadhesiveness and an increased gastric residence time, thus enhancing their oral bioavailability [29,31]. Interestingly, lipid-based LLCs of piperine, a weakly hydrophobic drug (solubility in water of 4 × 10^4^ ng/mL) [19], hydrophilic drugs (glucose, Allura Red, and FITC-dextrans) [58], and amphiphilic drugs (amphotericin B) were loaded in cubosomes as sustained-released oral formulations with improved bioavailability. Yang et al. explained that the enhanced solubility and gastrointestinal permeability of their amphotericin B-loaded cubosomes were because of their enhanced lymphatic drug transport and the fluidity of the enterocyte membrane [17]. Cubosomes containing glyceryl monooleate are widely accepted as candidates for lymphatic absorption via the chylomicron pathway after an oral administration.

### 3.2. Targeting the M Cell Pathway

Polysaccharide, an immunostimulant, was incorporated into cubosomes. The cubosomes were reported to have a higher immune activity compared to that of cubosomes or polysaccharides alone. The enhanced immune responses were explained by the promotion of antigen transport into draining lymph nodes and the efficient dendritic cell activation and memory T-helper cell differentiation in the draining lymph nodes [59]. M cells are mucosal epithelial cells specialized to transport antigens across the mucosa; therefore, particles can be decorated with ligands to mimic the transportation of antigens through the M cell pathway [5,7,9,60–62]. The intestinal M cell pathway is important for the uptake of proteins and peptides, which are nearly impossible to absorb through the intestinal epithelium.

Particle size influences the in vivo fate of particles and their ex vivo cellular interaction [63]. While smaller sized lipophilic LLCs can stay longer in the gastrointestinal tract due to their enhanced intestinal permeability, particles with size values of 550 nm and larger are preferred to be transported via the lymphatics [64], and microparticles (particle size of 1–5 μm) are often trapped in Peyer’s patches, thus activating the immune response [5]. Optimal particle surface hydrophobicity was also investigated. Hydrophobic particle surfaces that bind to proteins in the intestinal fluid can result in higher M cells transport efficiency for the particles [5]. Zeta potential values of the particles, indicating the particles’ stability, should be optimized in the development of micro/nanoparticles that target the M cell pathway. The ligand-conjugated nanoparticles (P2Ns–gambogic acid) targeting CD71s synthesized by Ganugula et al., polymeric-coated microparticles prepared by Shakweh et al., chitosan-modified solid lipid nanoparticles prepared by Shi et al., and chitosan-coated liposomes prepared by Channarong et al. successfully accumulated in Peyer’s patches due to their macromolecular size and lipophilicity [65–68]. These studies demonstrated the translational potential of lipid-based, polymeric, and hybrid polymer-lipid nanoparticles that can target lymphatic tissues through oral administration. Additionally, results showed that rod-like (hexagonal, cylindric) and disk-like particles had a longer retention time in the gastrointestinal tract with a higher penetration and achieved a higher lymphatic transport compared to spheres [63,69]. Overall, we can conclude that LLCs, the lipid-based, multilayered, hexagonal (rod-), and/or cubosomal (cubic) colloidal vesicles, can be conjugated and/or coated with other lipids, polymers, peptides, and proteins to form complex nanocomposites that target the lymphatic M cell pathway.

## 4. Facilitating Oral Drug and Vaccine Delivery using LLC Systems with Multiple Lymphatic Transport Mechanisms

Models mimicking the function of either chylomicrons or M cells have been developed for the evaluation of lymphatic transport. Caco-2 cell permeability assay has been established to measure the rate of flux of a drug across polarized human colon epithelial cell lines (monolayers) [50]. The Ussing chamber system has been used to measure intestinal permeability and the transport of drugs across the epithelial tissues [70]. Tissues from the ileum were isolated after the oral administration of bile-acid-conjugated solid nanoparticles and observed with confocal microscopy to visualize the enterocytes. Bile acid transporter was confirmed to mediate cellular uptake and chylomicron transport pathways [71,72]. Although chylomicron association predictive models were established [73] and permeability assays using Caco-2 cell lines with specific M cell-mimicking capacities were performed [74], lymphatic transport mechanisms could not be effectively clarified. When the uptake of particles through Peyer’s patches was higher, the enhanced absorption mechanisms of particles were related to the M cell pathway but were not limited to that. One of the goals of encapsulating therapeutics into LLCs is to target the lymphatic transport, but it was difficult to refer to the lymphatic transport with some LLC formulations, even though their intestinal permeability was enhanced. Usually, drugs transported to lymph should be highly lipophilic and should be associated with chylomicron. M cell pathway and other mechanisms of lymphatic transport can co-occur in the cases that hydrophilic drug-loaded LLCs cannot be taken up into epithelial cells as intact particles [17,19,75]. Multiple mechanisms of lymphatic transport including M cells, chylomicrons, and transcellular and paracellular pathways at the tight junction of epithelial cells have been reported [65,67,76]. We propose that lipid-based LLCs having chylomicron-binding capacity may take advantage of the chylomicrons to be delivered. Furthermore, the conjugation of LLCs with an antigen, a polymer, or a virus with proper chain length, molecular weight, and optimal proportions may help trigger the immune system. We recommend targeting different pathways and using different transport mechanisms to enhance lymphatic transport and oral bioavailability.

## 5. Conclusions

The oral route is one of the most common routes of drug administration, yet it remains a significant delivery challenge. The physiology of the gastrointestinal tract is complex. Overcoming the first-pass metabolism by targeting the intestinal lymphatic system with lipid nanoparticles, especially LLCs, can be a solution. Firstly, we characterized and described the unique structures of LLC phases, which influence the drug encapsulation, localization, and sustained-release behavior of LLCs. Secondly, LLCs can be engineered with bioactive lipids and polymers to increase their stability, optimize their particle shape, and size, and, ultimately, affect the intestinal lymphatic pathways. We recognize these LLC nanoplatforms as a promising generation of smart drug delivery systems for oral administration that can achieve a good lymphatic targeting efficacy while protecting the drug from the gastrointestinal environment.

## Figures and Tables

**Table 1.** The compositions and phases of LLC systems.

Water Phase	Lipid Phase	Surfactant	Mesophase	References
low water content	glycerol monooleate, HLB = 4.2, forms micellar solution with water above CMC of 4 × 10 ^−6^	Pluronic^®^ F127	bicontinuous cubic phase (V_2_) *Ia3d* or *Im3m*	[26–30]
high water content			bicontinuous cubic phase (V_2_) *Pn3m*	[17,26–28]
phosphate-buffered saline (PBS)	phytantriol, HLB = 3.8	Pluronic^®^ F127	bicontinuous cubic phases (V_2_) with the *Pn3m* or *Im3m* space groups	[30,31]
water	phytantriol and oleic acid	Pluronic^®^ F127	reverse hexagonal (H_2_)	[25,29]
water	selachyl alcohol (glyceryl monooleyl ether)	Pluronic^®^ F127	reverse hexagonal phase (H_2_)	[29]
	oleyl glycerate, HLB = 3.5		reverse hexagonal phase (H_2_)	

**Table 2.** Advantages and limitations of techniques used to analyze LLC mesophases.

Techniques		Advantages	Limitations	References
Polarized optical microscopy		The most common tool to characterize liquid crystals. The original method for characterizing thermotropic mesophases. It detects the existence of liquid crystal phases in a solution.	Only allows the characterization of the pre-determined phase, because different phases are defined by their order, which must be observed.	[22,32,40–42]
Thermal optical microscopy, DSC with polarized optical microscopy		Determine the thermal stability, phase transitions, transition enthalpies, phase sequences, temperature dependence of spontaneous polarization, and switching time.		
Scanning electron microscopic (SEM)		Creates an image of the shape and surface of freeze-dried samples by detecting reflected electrons. SEM photographs detect possible morphological changes that occurred during sample treatment.	Dehydration Only sees the fractured surfaces of the treated samples.	[22,43,44]
TEM		The structural details of the sample can be seen by transmitted electrons passing through the sample. Cryo-EM is where the TEM samples is studied at cryogenic temperatures.	Various TEM sample preparation techniques with multiple steps including cutting, fixation, filtration, staining, and dehydration.	[18–20,22,25,29,37]
SAXS	Small-angle (2θ < 5°) (SA) X-ray scattering and/or neutron scattering are optimal tools to study LLC particles of 1–100 nm size.	Allow the measurements of size, shape, separation, and interactions between the scattering particles. The scattering of X-rays by electrons of the atoms in a crystal lattice is determined, thus leading to the identification of the space group related to the crystal structure of the sample.	The sequence of mesophases is evident (phase transition). Experiments are often run at various concentrations of mesogen. Mesophases must be in equilibrium and form colloidal dispersions stability.	[17,18,25,37,38,45]
SANS		Compared to SAXS, SANS is non-destructive and is a contrast variable. Neutron scattering can detect the exact location of movable monomers and crosslinkers within the sample.		

## Data Availability

The authors confirm that the data supporting this study are within the articles.
